# Bioinorganic Chemistry in Thyroid Gland: Effect of Antithyroid Drugs on Peroxidase-Catalyzed Oxidation and Iodination Reactions

**DOI:** 10.1155/BCA/2006/23214

**Published:** 2006-11-12

**Authors:** Gouriprasanna Roy, G. Mugesh

**Affiliations:** Department of Inorganic and Physical Chemistry, Indian Institute of Science, Bangalore 560 012, India

## Abstract

Propylthiouracil (PTU) and methimazole (MMI) are the most commonly used antithyroid drugs. The available data suggest that these drugs may block the thyroid hormone synthesis by inhibiting the thyroid peroxidase (TPO) or diverting oxidized iodides away from thyroglobulin. It is also known that PTU inhibits the selenocysteine-containing enzyme ID-1 by reacting with the selenenyl iodide intermediate (E-SeI). In view of the current interest in antithyroid drugs, we have recently carried out biomimetic studies to understand the mechanism by which the antithyroid drugs inhibit the thyroid hormone synthesis and found that the replacement of sulfur with selenium in MMI leads to an interesting compound that may reversibly block the thyroid hormone synthesis. Our recent results on the inhibition of lactoperoxidase (LPO)-catalyzed oxidation and iodination reactions by antithyroid drugs are described.

## INTRODUCTION

**Figure 1:**
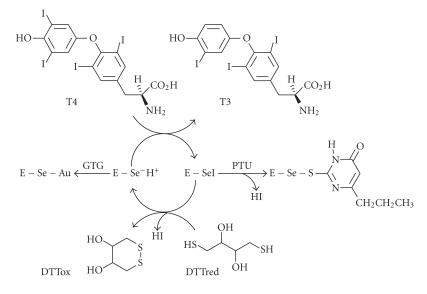
Proposed mechanism for the deiodination of thyroxine by ID-I and inhibition of ID-I by *n*-propyl-2-thiouracil (PTU) and gold thioglucose (GTG).

Thyroxine or 3,3′,5,5′-tetraiodothyronine (T4) is the major hormone secreted by the follicular cells of the thyroid gland. This hormone is produced on thyroglobulin by thyroid peroxidase (TPO)/hydrogen peroxide/iodide system. The synthesis of T4 by TPO involves two independent steps: iodination of tyrosine and phenolic coupling of the resulting iodotyrosine residues [1–5]. The prohormone T4 is then converted to its biologically active form T3 by an outer ring deiodination pathway. This particular reaction is catalyzed by a selenocysteine-containing enzyme called iodothyronine deiodinase (ID-I), which is present in highest amounts in liver, kidney, thyroid, and pituitary [6–16]. The thyroid gland also produces an inactive metabolite rT3 by an inner ring deiodination pathway. The triiodo derivatives T3 and rT3 are further metabolized by inner ring and outer ring deiodination, respectively, by ID-I, ID-II, and ID-III to produce the inactive metaboilite T2 (3,3′- T2, 3,5- T2, and 3′,5′- T2). The outer ring 5′-deiodination catalyzed by the ID-I enzyme is considered to be the first step in thyroid hormone action because this is the only deiodination pathway that leads to the formation of an active thyroid hormone. It is now widely accepted that the deiodination catalyzed by ID-I is a ping-pong, bisubstrate reaction in which the selenol (or selenolate) group of the enzyme (E-SeH or E-Se^−^) first reacts with thyroxine (T4) to form a selenenyl iodide (E-SeI) intermediate. Subsequent reaction of the selenenyl iodide intermediate with an as yet unidentified intracellular cofactor completes the catalytic cycle and regenerates the enzyme active site (Figure 1) [8, 14]. Although it is customary to use dithiothreitol (DTT, 1,4-dithiothreitol, *Cleland's reagent*) as the second substrate in in vitro experiments, the identity of the physiological second substrate is still uncertain. The tripeptide glutathione (GSH) can also act as a thiol cosubstrate, but GSH is a much less potent cofactor than DTT for ID-I [10, 17]. In addition to GSH, other native thiols such as dihydrolipoic acid or dihydrolipoamide may serve as cofactors for ID-I [17, 18]. Therefore, Figure 1 may be an incomplete or incorrect representation of the catalytic mechanism of ID-I since evidence for the cofactor systems mentioned above has only been presented for in vitro studies and not for in vivo analysis.

**Figure 2:**
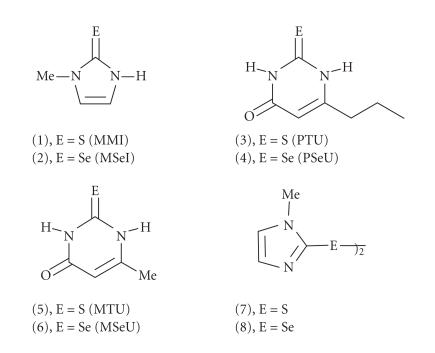
Chemical structures of some commonly employed antithyroid drugs and their selenium analogues.

Although the deiodination reactions are essential for the function of thyroid gland, the activation of thyroid stimulating hormone (TSH) receptor by autoantibodies leads to an overproduction of thyroid hormones. As these antibodies are not under pituitary feedback control system, there is no negative influence on the thyroid activity and, therefore, the uncontrolled production of thyroid hormones leads to a condition called “hyperthyroidism.” The overproduction of T4 and T3 can be controlled by blocking the thyroid hormone biosynthesis or reducing the conversion of T4 to T3. The thiourea drugs such as methimazole (1, MMI), 6-*n*-propyl-2-thiouracil (3, PTU), and 6-methyl-2-thiouracil (5, MTU) are generally employed for this purpose (Figure 2). Despite the importance of these antithyroid drugs in the treatment of hyperthyroidism, the detailed mechanism of their action is still not clear. According to the initially proposed mechanism, these drugs may divert oxidized iodides away from thyroglobulin by forming stable electron donor-acceptor complexes with diiodine, which can effectively reduce the thyroid hormone biosynthesis [19, 20]. It has also been proposed that these drugs may block the thyroid hormone synthesis by coordinating to the metal center of thyroid peroxidase (TPO) [21]. After the discovery that the ID-I is responsible for the activation of thyroxine, it has been reported that PTU, but not MMI, reacts with the selenenyl iodide intermediate (E-SeI) of ID-I to form a selenenyl sulfide as a dead end product, thereby blocking the conversion of T4 to T3 during the monodeiodination reaction (Figure 1) [8, 14, 22–24]. The mechanism of antithyroid activity is further complicated by the fact that the gold-containing drugs such as gold thioglucose (GTG) inhibits the deiodinases by reacting with the selenol (or selenolate) group of the native enzyme (Figure 1) [8, 24].

In recent years, the selenium analogues 2 (MSeI), 4 (PSeU), and 6 (MSeU) attracted considerable attention because these compounds are expected to be more nucleophilic than their sulfur analogues and the formation of an −Se−Se− bond may occur more readily than the formation of an −Se−S− bond with the ID-I enzyme [25–29]. However, the data derived from the inhibition of TPO by selenium compounds show that these compounds may inhibit the TPO activity by a different mechanism. We have recently shown that the unexpected behavior of the selenium compound MSeI as compared to that of its sulfur analogue may be due to the existence of this compound in the zwitterionic form and its facile oxidation to the corresponding diselenide (8) [30, 31]. In this paper, we summarize our recent results on the effect of antithyroid drugs on peroxide-catalyzed oxidation and iodination reactions. In addition, we show that the replacement of sulfur with selenium in MMI leads to an interesting compound (MSeI) that exhibits significant glutathione peroxidase (GPx)-like antioxidant activity.

## INHIBITION OF LACTOPEROXIDASE-CATALYZED OXIDATION BY ANTITHYROID DRUGS

**Scheme 1:**
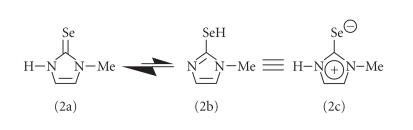
The possible tautomeric structures of compound 2. The compound exists predominantly in its zwitterionic form 2c, which may have a partial C−Se double bond character.

The effect of antithyroid drugs on peroxidase-catalyzed oxidation was studied in vitro by using spectroscopic techniques. The enzyme inhibition experiments were carried out with iron-containing lactoperoxidase (LPO) since it is readily available in purified form. Furthermore, LPO has been shown to behave very similarly to TPO with respect to oxidation of organic substrates and iodination of thyroglobulin and other iodide acceptors [32]. We have employed 2,2′-azio-bis-3-ethyl-benthiazoline-6-sulfonic acid (ABTS) and H_2_O_2_ as substrates [33] to determine the half-maximal inhibitory concentration (IC_50_) of test compounds. The IC_50_ values for the inhibition of LPO-catalyzed oxidation of ABTS by 1–3 and 5 are summarized in Table 1 [31]. The sulfur compound MMI inhibited the LPO activity with an IC_50_ value of 7.0 ± 1.1 *μ*M, which is much lower than those observed with PTU and MTU. The selenium analogue (2) also inhibited LPO activity and the IC_50_ value was found to be almost 2-3 times lower than those of PTU and MTU. The higher activity of MMI as compared with those of PTU and MTU is in agreement with the previous studies on the inhibition of TPO. Since the activation of the iron center in TPO must proceed through an interaction of Fe(III) with H_2_O_2_, TPO inactivation may occur through a competitive coordination of the drug to iron, assisted by hydrogen bonding with a histidine residue of the TPO enzyme [34]. Under these conditions, MMI might compete more successfully than PTU with H_2_O_2_ because the hydrogen-bond (hard) basicity p*k*_HB_ value of MMI (2.11) is much higher than that of PTU (∼ 1.32). Similarly to PTU, the methyl derivative 5 is also expected to be a weak inhibitor of TPO. On the other hand, compound 2, which exists predominantly in the zwitterionic form (Scheme 1) [35], probably does not have the ability to coordinate to the iron center; therefore, this compound may inhibit the LPO activity by a different mechanism. Although the zwitterionic selenolate can be oxidized in solution to give the corresponding diselenide (8), the observed inhibitory activity must be ascribed entirely to the presence of the reduced form (2) as the oxidized compound (8) does not show any noticeable inhibition behavior under identical experimental conditions.

**Figure 3:**
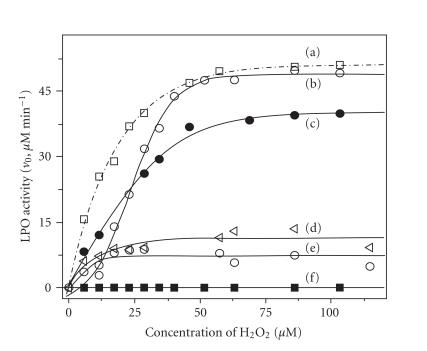
Plot of initial rates (v_o_) for the LPO-catalyzed oxidation of ABTS versus concentration of H_2_O_2_: (a) control activity; (b) 40 *μ*M of 2; (c) 40 *μ*M of 8; (d) 80 *μ*M of PTU; (e) 80 *μ*M of MTU; (f) 40 *μ*M of MMI. Conditions: LPO: 6.5 nM; H_2_O_2_: 22.9 *μ*M (see [31]).

Taurog et al have shown that MMI and related derivatives irreversibly inhibit LPO and TPO, leading to a complete inactivation of the enzymes [36–39]. Doerge and others have shown that mammalian peroxidases including LPO may activate the antithyroid drugs through S-oxygenation to produce the corresponding sulfoxides or sulfinic acids [40, 41]. They have also shown that the suicide inactivation of LPO and TPO by MMI proceeds through the S-oxygenation of the thione moiety to form a reactive sulfinic acid, which binds covalently to the prosthetic heme and irreversibly blocks enzyme activity [42–45]. Given the higher reactivity of selenium compounds as compared with the sulfur derivatives toward oxidation, it is possible that the facile oxidation of the selenium compounds may lead to an efficient inhibition of LPO activity. With this in mind, we treated MMI, PTU, MTU, and MSeI with H_2_O_2_ before adding LPO and ABTS. The LPO activity was measured several times by increasing the time for the reaction of the test compounds with H_2_O_2_. Remarkably, MSeI (2) inhibited the enzyme within few seconds even at lower concentrations, which can be ascribed to the facile oxidation of the reactive selenolate group in 2 (MSeI) by H_2_O_2_ or by the oxidized enzyme. Because MMI also inhibits the enzyme very efficiently, we have carried out further experiments to prove that the mechanisms by which MMI and MSeI exert their inhibitory action are different. The initial rates (v_0_) derived from various concentrations of H_2_O_2_ were plotted against the concentration of H_2_O_2_. The LPO activity was completely inhibited by 40 *μ*M MMI, and the enzyme's activity could not be recovered by increasing the H_2_O_2_ concentration (Figure 3f) [31]. The LPO activity could not be recovered even at lower concentration of MMI (10 *μ*M) and higher concentration of H_2_O_2_ (230 *μ*M). This suggests that MMI does not act on H_2_O_2_ but acts on the enzyme itself, leading to an irreversible inhibition as previously proposed. On the other hand, 2 also inhibited the LPO activity as efficiently as MMI, but in this case, the enzyme's activity could be completely recovered by increasing H_2_O_2_ concentration (Figure 3b). These observations may support the assumption that MSeI, in contrast to MMI, does not interfere with the native enzyme directly but it inhibits the LPO activity by reducing the H_2_O_2_, which is required for the oxidation of the iron center in LPO. The reduction of H_2_O_2_ by 2 may become more efficient in the presence of suitable thiols such as GSH because this process may constitute a redox cycle involving a catalytic reduction of H_2_O_2_ (glutathione peroxidase (GPx) activity) [46]. Thus, compound 2 mimics the action of GPx, a selenoenzyme that protects the cellular components from oxidative damage by reducing H_2_O_2_ with the help of GSH [16]. Recently, the GPx enzyme present in thyroid gland has been shown to inhibit the iodination reactions by degrading the intracellular H_2_O_2_ [47, 48]. The high GPx activity of the key compound 2 leads to an assumption that the antithyroid drugs may act as antioxidants in addition to their inhibition behavior.

## INHIBITION OF LACTOPEROXIDASE-CATALYZED IODINATION BY ANTITHYROID DRUGS

**Scheme 2:**
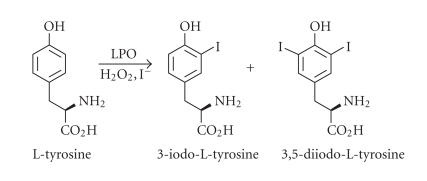
Iodination of L-tyrosine by LPO/peroxide/iodide system.

The interesting results that we obtained from the inhibition of LPO-catalyzed oxidation reactions by MSeI (2) prompted us to study the effect of this compound and related derivatives on the LPO-catalyzed iodination reactions [49]. In addition, we have studied the reactivity of MSeI toward iodine because the effect of the selenium compounds on the iodination of tyrosine and the identification of the products formed in the reactions of these compounds with iodine are crucial in understanding the mechanism of action in vivo of these drugs. The iodination of tyrosine was studied by using LPO/ H_2_O_2_/I^−^ assay and the initial rates for the conversion of L-tyrosine to 3-iodo L-tyrosine (Scheme 2) were determined by an HPLC method.

**Figure 4:**
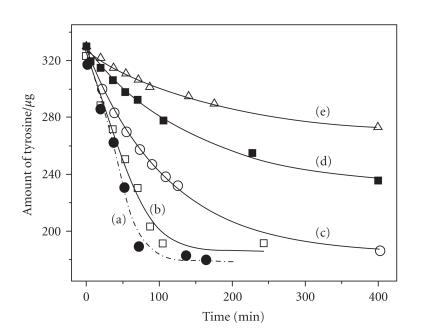
Inhibition of the LPO-catalyzed iodination of L-tyrosine by MSeI. The decrease in the amount of tyrosine with time was followed by HPLC: (a) control; (b) 6 *μ*M of 2; (c) 9 *μ*M of 2; (d) 12 *μ*M of 2; (d) 15 *μ*M of 2; and (e) 20 *μ*M of 2 (see [49]).

As the formation of 3,5-diiodo-L-tyrosine was also observed in the reaction, only the initial 5%–10% of the conversion was followed where only a trace amount of the diiodo compound was produced. The decrease in the concentration of L-tyrosine was followed by measuring the peak area at 277 nm and the amount of tyrosine present in the solution at a given time was calculated from the calibration plot obtained by injecting known concentrations of L-tyrosine. The effect of compound 2 on the iodination reaction was determined at various concentrations of 2 under identical experimental conditions (Figure 4). The incubation mixtures for the HPLC analysis contained KI, L-tyrosine, hydrogen peroxide, and LPO enzyme. The mixture was incubated in phosphate buffer at room temperature and aliquots were injected onto the HPLC column and eluted with gradient solvent system (0.1% TFA in water-MeCN). The decrease in the amount of tyrosine (*μ*g) was calculated from the calibration plot. The concentration of compound 2 was varied from 6 *μ*M to 20 *μ*M, and a significant inhibition was observed even at the lowest concentration of 2.

**Figure 5:**
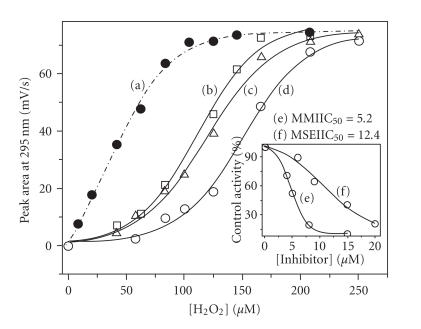
Inhibition of the LPO-catalyzed iodination of L-tyrosine by MSeI. Effect of H_2_O_2_ on the inhibition by 2; (a) 0 *μ*M; (b) 20 *μ*M; (c) 30 *μ*M; (d) 40 *μ*M; *inset*: inhibition of tyrosine iodination by (e) 1; (f) 2 at a fixed H_2_O_2_ concentration.

To understand the effect of peroxide substrate on the reaction rate and the inhibition, the LPO activity was determined at various concentrations of hydrogen peroxide. In addition, the effect of peroxide on the inhibition of LPO-catalyzed iodination by antithyroid drugs 1 and 2 was evaluated by carrying out the experiments at various concentrations of H_2_O_2_. In this HPLC assay, the incubation mixtures containing KI, L-tyrosine, LPO enzyme, and various concentrations of hydrogen peroxide were incubated at room temperature. The aliquots were removed from the reaction mixture at various time intervals, injected onto the HPLC column and eluted with gradient solvent system (0.1% TFA in water-MeCN). The formation of monoiodo tyrosine was followed at 295 nm. The initial rates (v_0_) derived for various concentrations of H_2_O_2_ were plotted against the concentration of H_2_O_2_. Although the LPO activity was inhibited by 2 at lower concentrations of H_2_O_2_, the enzyme's activity could be completely recovered by increasing H_2_O_2_ concentration (Figure 5). These results suggest that the concentration of H_2_O_2_ has a dramatic effect on the inhibition of iodination reaction by compound 2 (Figure 5) [49].

The IC_50_ values for the inhibition of LPO-catalyzed iodination of L-tyrosine by the test compounds were also determined by following the same procedure. The initial rates for the iodination reaction were determined at various concentrations of inhibitors. The inhibition curves obtained by plotting the percentage control activity against the concentration of inhibitors are shown in Figure 5 (*inset*). As expected, MMI exhibited a strong inhibition with an IC_50_ value of 5.2 *μ*M, which is comparable with the IC_50_ value obtained for the LPO-catalyzed oxidation reaction [30, 31]. The selenium analogue (2) also showed a strong inhibition with an IC_50_ value of 12.4 *μ*M, which is consistent with the effect of this compound on peroxidase-catalyzed oxidation reactions [30, 31]. Similarly to the LPO-catalyzed oxidation of 2,2′-azino-bis-3-ethylbenz-thiazoline sulfonic acid (ABTS), this suggests that the selenium analogue may inhibit the LPO by a different mechanism. The diselenide 8, on the other hand, did not show any noticeable inhibition under identical conditions. This confirms that the reduction of the diselenide to the corresponding selenolate is essential for an efficient inhibition of LPO-catalyzed oxidation or iodination reactions.

## INTERACTION OF ANTITHYROID DRUGS WITH IODINE

**Figure 6:**
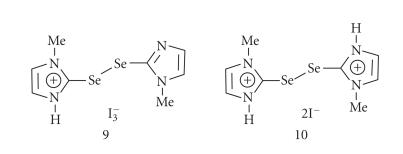
Chemical structures of compounds 9 and 10 derived from the compound 2.

**Figure 7:**
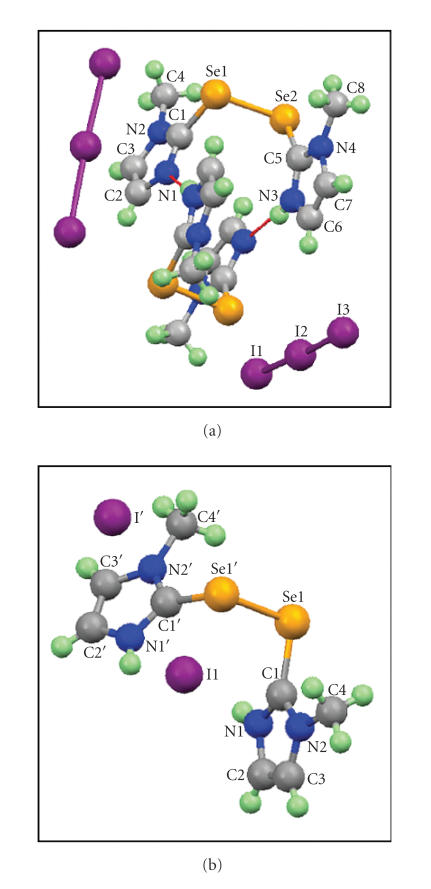
Molecular structures of (a) monocation 9 showing the hydrogen bonding between two monocations and (b) dication 10 [49].

The nature of charge-transfer complexes between heterocyclic antithyroid drugs and diiodine is an important area of interest in the study of hyperthyroidism [50], and the electrical properties in general and the superconducting ability, in particular, of sulfur-iodine complexes are also of current interest [51–54]. Recently, a great deal of effort has been devoted to the understanding of the interaction of antithyroid drugs with iodine [55–59]. These studies may provide insight into the nature of products formed during the inhibition of thyroid hormone synthesis. As mentioned in the introduction, it has been proposed that these drugs may divert oxidized iodides away from thyroglobulin by forming stable electron donor-acceptor complexes with diiodine, which can effectively reduce the thyroid hormone biosynthesis [19, 20]. Because the oxidation of MMI to the corresponding disulfide (7) by TPO/H_2_O_2_/I^−^ system is associated with the reaction of MMI with I_2_ [55–59], we have investigated the interaction of 2 and 8 with iodine. It has been reported that I_2_ chemically oxidizes MMI to produce ionic disulfides that exist in two different protonated forms [55]. It is unknown whether the selenium analogue of MMI, in its reduced form, also undergoes such oxidation by I_2_ to produce ionic species (Figure 6). Therefore, we carried out the experiments with the reduced species (2), which exists in its zwitterionic form [31, 35]. The reaction of 2 with I_2_ in CH_2_Cl_2_ produced red-brown crystals. Interestingly, the X-ray crystal structure shows the formation of compound 9, which consists of a monocation containing a diselenide and I_3_^−^ as counterion (Figure 7) [49]. This is in contrast to the reaction of MMI with I_2_ in CH_2_Cl_2_, which afforded a disulfide-containing dication and I_8_^−^ as counterions [55].

The formation of the monocationic species 9 is interesting from a chemical point of view as only one of the imidazole rings undergoes oxidation. It should be mentioned that the N-methylation on MMI has been shown to abolish its TPO inhibitory activity [60]. Freeman et al have shown that the reaction of the N-methylated derivative (1,3-dimethylimidazole-2-thione) with I_2_ does not produce any disulfide, but it produces a 1 : 1 thione : I_2_ charge-transfer adduct [61]. The N-methylated derivative of 2 (1,3-dimethylimidazole-2-selone), on the other hand, produces a hypervalent “T-shaped” compound having I−Se−I moiety [62, 63]. It should be noted that the reaction of the methylated analogue of 2, 1,3-dimethylimidazole-2-selone, with one equivalent bromine affords a hypervalent compound having Br−Se−Br moiety, whereas the corresponding reaction utilizing a half-equivalent bromine leads to the formation of a diselenide dication having two Br^−^ as counterions [64]. Therefore, the existence of 2 in its zwitterionic (or selenolate) form is probably responsible for its different reactivity toward iodine. Stable open-chain cationic diselenide species are very uncommon in the literature and to the best of our knowledge no structural information is available for complexes derived from the reactions of selenium analogues of antithyroid drugs with iodine. The chemical oxidation of 2 by I_2_ suggests that compound 8, which exists in the oxidized form of 2, may not produce any ionic species. To test this, the diselenide 8 was treated with I_2_ in a 1 : 2 molar ratio in CH_2_Cl_2_.

This reaction yielded a brown solution from which dark-brown crystals were obtained on standing at room temperature. Surprisingly, the X-ray crystal structure shows the formation of a monocationic species, which is identical with that obtained from the reaction of 2 with I_2_ (Figure 7). The formation of a cationic species in this reaction is quite unexpected because the reactions of iodine with diselenides generally produce selenenyl iodide species or charge-transfer complexes having diselenide-molecular iodine adducts [65]. It is also known that some of the selenenyl iodides may undergo disproportionation to give diselenide-iodine complexes.

**Figure 8:**
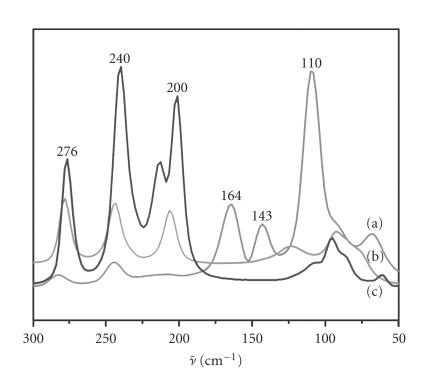
FT-Raman spectra of the monocation 9 (a), diselenide 8 (b), and the dication 10 (c) (see [49]).

The far-IR spectrum of complex 9 shows a distinct band at 135 cm^−1^ for the *ν*(I−I) stretching vibration mode. This is in agreement with the fact that I_2_ gives a strong band at 180 cm^−1^ in the solid state, which shifts to lower wavenumbers upon coordination to a donor atom, reflecting a reduction in the I−I bond order [56]. The FT-Raman spectrum of the complex in the *ν*(I−I) region shows intense peaks at 164 cm^−1^, 143 cm^−1^, and 110 cm^−1^. In addition, a weak band is observed around 67 cm^−1^ (Figure 8). The band at 110 cm^−1^ can be certainly assigned to the *ν*_1_ symmetric stretching of I_3_^−^, which being a symmetrical ion normally exhibits only one Raman active band. However, when a distortion of I_3_^−^ occurs, the antisymmetric stretching may become Raman active and additional bands at higher (140 cm^−1^–130 cm^−1^) and at lower frequencies (80 cm^−1^–70 cm^−1^) may be observed [56, 66]. Therefore, the relatively weak bands at 143 cm^−1^ and 67 cm^−1^ can be attributed to the antisymmetric stretching and deformation motions, respectively, for the I_3_^−^ ion (Figure 8a).

The single crystal X-ray studies confirm the proposed structure of 9 (Figure 7), which consists of two independent diselenide monocations [Se−Se: 2.382 Å; 2.364 Å]. These diselenide cations interact with their symmetry equivalents through N−H⋯N hydrogen bonds to form dimeric units with overall charge 2+. The charge balance in the crystals is achieved by the presence of two I_3_^−^ anions. The two C−Se bond lengths in each subunit are unequal due to the monoprotonation of the one of the five-membered rings [C−Se: 1.886–1.890 Å]. The I−I bond lengths observed also differ significantly from the corresponding I−I bond length of I_2_ in the solid state (2.715 Å). The two I−I bond lengths of the I_3_^−^ species in complex 9 range from 2.888 Å to 2.919 Å, indicating a slight distortion of the I_3_^−^ moiety. This distortion is probably responsible for additional bands in the FT-Raman spectrum of the complex.

In the reaction between 8 and I_2_ in dichloromethane, the concentrations of I_2_ do not appear to change the nature of products. During our attempts to oxidize the second ring using various concentrations of I_2_ up to an excess, only the monocation was obtained as a stable product. However, the choice of solvent has been found to have a large influence on the nature of products formed. The reaction of 8 with I_2_ in a 1 : 2 molar ratio in water produced a mixture containing both monocation (9) and dication (10) as confirmed by single-crystal X-ray studies (Figure 7). In contrast to the monocation, the charge balance in the crystal of dication is achieved by two I^−^ anions. In compound 10, the average C−Se bond length of 1.895 Å is comparable with that of the diselenide 8 (1.880 Å) [30], but this is significantly longer than the average C−Se bond length (1.848 Å) found in compound 2 that exists in a zwitterionic form [35]. As expected, the FT-Raman spectrum of compound 10 shows no peaks in the region of lower wavenumbers (Figure 8c), indicating the absence of any polyiodide species in the crystals.

## CONCLUSION AND OUTLOOK

Our recent results show that the selenium analogue of methimazole (MSeI) exists predominantly in its zwitterionic form, in which the selenium atom carries a negative charge and the five-membered heterocyclic ring carries a positive charge. In contrast to the sulfur analogue, the zwitterionic form of MSeI is unstable and oxidizes in air to the corresponding diselenide. The resulting diselenide can be easily reduced by reducing agents such as NaBH_4_ or glutathione (GSH). In its reduced form (zwitterionic or selenolate), MSeI effectively and reversibly inhibits the lactoperoxidase (LPO)-catalyzed oxidation reactions. These results suggest that MSeI may not interfere with the native enzyme directly, but it may inhibit LPO either by reducing the H_2_O_2_ that is required for the oxidation of the iron center in LPO or by interfering with the oxidized enzyme. In the presence of GSH, MSeI may constitute a redox cycle involving a catalytic reduction of H_2_O_2_ and thereby mimics the glutathione peroxidase (GPx) activity in vitro. In addition, MSeI effectively inhibits the LPO-catalyzed iodination of L-tyrosine and the inhibition could be completely recovered by increasing the H_2_O_2_ concentration. These studies reveal that the degradation of the intracellular H_2_O_2_ by the selenium analogues of antithyroid drugs may be beneficial to the thyroid gland as these compounds may act as antioxidants and protect thyroid cells from oxidative damage. In addition to its antioxidant activity, MSeI reacts with I_2_ to produce novel ionic diselenides containing iodide or polyiodide anions, which might be effective intermediates in the inhibition of thyroid hormones. However, further studies with TPO are required to derive some firm conclusions regarding the mode of action of the antithyroid drugs. Our future work will focus on the design and synthesis of novel sulfur and selenium compounds and study of their antithyroid and antioxidant activities.

## Figures and Tables

**Table 1:** Inhibition of LPO activity by 1–3, and 5 [31].

No	Compound	IC_50_ (*μ*M)^a^

1	MMI (1)	7.0 ± 1.1
2	MSeI (2)	16.4 ± 1.5
3	PTU (3)	45.0 ± 2.1
4	MTU (5)	47.8 ± 0.1

^a^ Concentration of the compound causing 50% inhibition. Each IC_50_ value was calculated from at least three independent experiments.
